# Impact of sleep quality on disease progression in early-stage amyotrophic lateral sclerosis

**DOI:** 10.3389/fneur.2025.1545463

**Published:** 2025-04-10

**Authors:** Gan Zhang, Yong Chen, Lu Tang, Linna Bai, Hui Zhang, Hong Liu, Dongsheng Fan

**Affiliations:** ^1^Department of Neurology, Peking University Third Hospital, Beijing, China; ^2^Beijing Key Laboratory of Biomarker and Translational Research in Neurodegenerative Diseases, Beijing, China; ^3^Key Laboratory for Neuroscience, National Health Commission/Ministry of Education, Peking University, Beijing, China

**Keywords:** non-motor symptoms, sleep quality, amyotrophic lateral sclerosis, ΔFS, neurofilament light chain

## Abstract

Non-motor symptoms are clinical manifestations of amyotrophic lateral sclerosis (ALS). However, few studies have examined these symptoms in patients with early-stage ALS. We conducted a cross-sectional study to explore non-motor symptoms in 69 patients with ALS within 18 months of disease onset. The Pittsburgh Sleep Quality Index (PSQI), the Epworth Sleepiness Scale (ESS), and the Hospital Anxiety and Depression Scale (HADS) were used to evaluate sleep quality, daytime sleepiness, and anxiety and depression, respectively. Differences in the abovementioned non-motor symptoms between ALS patients and age-/sex-matched caregivers were examined, and correlations between these symptoms and the clinical features of ALS were analyzed. Compared to caregivers, ALS patients were more likely to report poor sleep [odds ratio (OR) and 95% confidence interval (95% CI) = 2.664, 1.276–5.560; *p* = 0.009] and excessive daytime sleepiness (EDS) [OR and 95% CI = 5.135, 1.640–16.072; *p* = 0.005]. The PSQI scores in ALS patients correlated significantly with the disease progression rate [ΔFS = (48-score on the Amyotrophic Lateral Sclerosis Functional Rating Scale-Revised, ALSFRS-R)/disease duration] [β(95% CI) = 2.867 (0.397, 5.336), *p* = 0.024] and plasma neurofilament light chain (NfL) levels [β (95% CI) = 0.041 (0.012, 0.070), *p* = 0.008). Our results revealed that the patients with early-stage ALS experienced poor sleep quality and daytime sleepiness and suggested that low sleep quality may be related to more rapid disease progression. Confounders were not obvious in the early stage of ALS, and our results suggested that these symptoms may be related to more severe and extensive pathological changes in the central nervous system.

## 1 Introduction

Amyotrophic lateral sclerosis (ALS) is a neurodegenerative disease with an unknown etiology that primarily affects motor neurons in the motor cortex, brainstem, and spinal cord (1). Due to the prominent motor symptoms, ALS has long been considered a disease that specifically affects the motor system. However, with continued research on this disease, an increasing number of studies have shown that ALS also affects other systems and presents manifestations other than motor symptoms (2).

Non-motor symptoms can also affect ALS patients' quality of life (3). In this context, an increasing number of studies have focused on non-motor symptoms in ALS patients. The most prevalent non-motor symptoms associated with ALS include insomnia, excessive daytime sleepiness, anxiety, depression disorders, and cognitive and behavioral impairments (4–8). However, the frequency of non-motor symptoms reported in various studies varies significantly. ALS is a slow-progressing disease, and differences in the movement and respiratory function of the patients included in these studies may have significant effects on other aspects of the disease. The heterogeneity of ALS and the differences in evaluation methods used may also contribute to this phenomenon. Therefore, more comprehensive studies on the non-motor symptoms of ALS require detailed criteria for the inclusion of patients. Studies of this type, especially those involving patients with early-stage ALS, are lacking. We investigated sleep and emotion-related non-motor symptoms in ALS patients who had disease durations of 18 months or less using questionnaires to evaluate the occurrence of these symptoms at the early stage of the disease and analyze the associations between these symptoms and the main clinical features of ALS.

This study helps address the current lack of information about early non-motor symptoms of ALS. Since the impairment of motor function in ALS patients at the early stage of the disease is not serious and the changes in life status caused by the disease are not significant, our results provide further insights into whether the non-motor symptoms of ALS are influenced by factors other than motor symptoms.

## 2 Methods

### 2.1 Participants

We collected detailed clinical data from 69 adult patients who met the definite, probable, or laboratory-supported criteria for ALS, as described in the revised El Escorial criteria, during their routine visits to our ALS clinic and inpatient ward between September 2020 and September 2021 (9). Patients who met any of the following criteria were excluded: (1) a clear or suspicious family history of ALS, (2) the presence of other central nervous system diseases or mental illnesses, (3) an inability to provide complete and reliable information due to factors such as language barriers, and (4) refusal to participate in the study. The control group consisted of 69 caregivers of these patients; they were recruited from the families of the patients and matched for age and sex to those in the ALS group. Controls who met any of the following criteria were excluded: (1) a history of central nervous system disease or mental illness, (2) an inability to provide complete and reliable information, and (3) refusal to participate in the study. It is crucial to emphasize that caregivers of ALS patients often experience compromised health status, including potential sleep disturbances and psychological distress, resulting from both the patient's condition and the inherent stressors associated with caregiving responsibilities—factors that may potentially confound our research findings.

### 2.2 Information collection

#### 2.2.1 Epidemiology and disease information

The sex, age, and body mass index (BMI) of the participants were recorded. We also collected the following parameters for each patient with ALS included in the study: (1) disease duration (months); (2) site of disease onset (bulbar or limb); (3) forced vital capacity as a percentage of the estimated value (FVC% pred); (4) disease stage according to the King's College ALS staging system (KCSS) (10); (5) Amyotrophic Lateral Sclerosis Functional Rating Scale-Revised (ALSFRS-R) score (11); and (6) disease progression rate: ΔFS = [48-ALSFRS-R total/disease duration (months)] (12).

#### 2.2.2 Sleep quality

To collect information on sleep quality, the Pittsburgh Sleep Quality Index (PSQI) was administered to the study participants (13). This questionnaire is widely used to quantitatively assess sleep quality in various groups of people and in the diagnosis of insomnia. The questionnaire includes seven components; each component is scored on a scale of 0–3 points, and the scores for each component are summed to obtain the total PSQI score. The higher the score, the worse the sleep quality. A total PSQI score > 5 was considered to indicate poor sleep. The results reflect the sleep quality of the participants during the month prior to the evaluation. The PSQI was evaluated one-on-one by a single clinician who specializes in sleep disturbances.

#### 2.2.3 Daytime sleepiness

The degree of daytime sleepiness was evaluated using the Epworth Sleepiness Scale (ESS) (14). This scale evaluates sleepiness in eight different scenarios, each of which includes four options: never dozing off (0 points), rarely dozing off (1 point), sometimes nodding off (2 points), and frequently dozing off (3 points). The scores for each scenario are summed to obtain the total score (0–24 points); higher scores indicate greater degrees of daytime sleepiness. We considered a total ESS score ≥10 to be indicative of excessive daytime sleepiness (EDS). The ESS scores were evaluated one-on-one by a single clinician specializing in sleep disturbances.

#### 2.2.4 Depression and anxiety

The Hospital Anxiety and Depression Scale (HADS), which is mainly used to screen for anxiety and depression in hospitalized patients, was used to evaluate the anxiety and depression levels of the study participants (15). The scale includes 14 questions: seven are used to assess anxiety and the other seven are used to assess depression. A total HADS score ≥ 8 for anxiety/depression was considered to indicate possible or definite anxiety/depression.

### 2.3 Measurement of plasma biomarkers

Peripheral plasma samples from 40 patients were collected in ethylenediaminetetraacetic acid (EDTA) citrate vacutainer tubes, centrifuged using a tabletop centrifuge, aliquoted, and stored at −80°C according to standard protocols until further analysis. Plasma biomarkers were measured using the single-molecule array (Simoa) platform (Quanterix Corp., MA, USA) for neurofilament light chain (NfL) at the laboratory of Wayen Biotechnologies, Inc., Shanghai, China.

### 2.4 Statistical analysis

The Kolmogorov–Smirnov test was performed to assess the normality of the data. Continuous data obtained from ALS patients and controls were compared using either an independent samples *t*-test (data distribution conforming to normality) or a rank sum test (data distribution not conforming to normality). The chi-squared test was performed to compare the classified data. Binary logistic regression analysis was performed to assess the associations between ALS and non-motor symptoms. This methodology was subsequently utilized to perform comparative analyses across two distinct dimensions: first, to examine non-motor symptom differences between the male and female ALS patients and second, to assess non-motor symptom variations between the male and female caregivers. In addition, multiple logistic regression analyses were conducted to evaluate the association between non-motor symptom profiles and different stages of the KCSS in ALS patients. Multiple linear regression analysis was performed to assess the associations between non-motor symptoms and other ALS features. Continuous variables were expressed as mean ± standard deviation (data distribution conforming to normality) or as median and quartile spacing (data distribution not conforming to normality), and categorical variables were expressed as specific values and proportions (n, %). The results of the binary logistic regression and multiple logistic regression analyses were expressed as odds ratios (ORs) with 95% confidence intervals (95% CIs), and the results of the multiple linear regression were expressed as β values with 95% CIs. All analyses were considered statistically significant at a p-value of < 0.05 (bilateral). All the statistical analyses were performed using SPSS V.25.0 (IBM, Armonk, NY, USA).

## 3 Results

### 3.1 Demographic and clinical information of the ALS patients and controls

A total of 69 patients with ALS and 69 control individuals were included in the study. There was no difference in age or sex between the ALS patients and controls. The mean BMI of the ALS patients was lower than that of the controls. The clinical characteristics of the study population, including the site of disease onset, disease duration, ALSFRS-R scores, ΔFS values, FVC% pred (available for 60 patients), and the distribution of patients according to the KCSS classification, are presented in Table 1.

**Table 1:** Demographic and clinical information and non-motor features of the patients with ALS and their caregivers.

**Demographic/clinical information/non-motor features**	**Patients with ALS (*n* = 69)**	**Controls (*n* = 69)**	***P*-value**
Age (years)	54.88 ± 10.91	53.03 ± 9.71	0.293
Sex (*n* of male participants/%)	44/63.8	35/50.7	0.121
BMI	23.63 ± 2.89	24.92 ± 3.00	0.011
Disease duration (months)	10.27 ± 3.85	/	/
Bulbar onset (*n*/%)	10/14.49	/	/
FVC% pred^*^	81.41 ± 20.29	/	/
ALSFRS-R score	42.00 [39.00, 45.00]	/	/
ΔFS	0.53[0.37, 0.83]	/	/
KCSS (*n*/%)			
1	26/37.68	/	/
2	29/42.03	/	/
3	12/17.39	/	/
4	2/2.90	/	/
Pittsburgh Sleep Quality Index score	6.00 [4.00, 9.50]	5.00 [4.00, 6.00]	0.035
Subjective sleep quality	1.00 [0.50, 2.00]	1.00 [1.00, 1.00]	0.393
Sleep latency	1.00 [0.00, 2.00]	1.00 [0.00, 1.00]	0.168
Sleep duration	1.00 [0.00, 2.00]	1.00 [0.00, 1.00]	0.078
Habitual sleep efficiency	1.00 [0.00, 1.50]	0.00 [0.00, 1.00]	0.002
Sleep disturbances	1.00 [1.00, 1.00]	1.00 [1.00, 1.00]	0.369
Use of sleep medication	0.00 [0.00, 0.00]	0.00 [0.00, 0.00]	0.709
Daytime dysfunction	1.00 [0.00, 2.00]	1.00 [0.00, 2.00]	0.467
Poor sleeper (*n*/%)	38/55.1	22/31.9	0.006
Epworth Sleepiness Scale score	5.00 [3.00, 9.00]	3.00 [2.00, 6.00]	0.043
EDS (*n*/%)	16/23.2	5/7.2	0.009
HADS-depression score	6.00 [3.00, 8.00]	5.00 [2.00, 7.00]	0.296
Doubtful or definite depression (*n*/%)	22/31.9	17/24.6	0.345
HADS-anxiety score	5.00 [3.00, 9.00]	5.00 [3.00, 7.50]	0.225
Doubtful or definite anxiety (*n*/%)	18/26.1	17/24.6	0.845

Independent samples t-tests or rank sum tests were used to compare continuous data, and chi-squared tests were used to compare classified data. The results are presented as mean ± standard deviation (t-tests), median [Q1, Q3] (rank sum tests), and specific values and proportions (chi-squared tests). ALS: amyotrophic lateral sclerosis; BMI: body mass index; FVC% pred: forced vital capacity as a percentage of the estimated value; ALSFRS-R: ALS Functional Rating-Revised; ΔFS = (48-ALSFRS-R score)/disease duration; KCSS, King's College staging system; EDS, excessive daytime sleepiness; HADS, Hospital Anxiety and Depression Scale; Q1, first quartile; Q3, third quartile. ^*^FVC% pred data were available for sixty patients with ALS.

### 3.2 Non-motor features of ALS patients and controls

The results of the rank sum test suggested that the total PSQI score of the ALS patients was greater than that of the controls (*p* = 0.035). The difference between the two groups was mainly observed in the habitual sleep efficiency subindex (*p* = 0.002). ALS patients showed no significant differences compared to the controls in their scores for subjective sleep quality, sleep latency, sleep duration, sleep disturbances, use of sleeping medication, or daytime dysfunction (*p* > 0.05). The chi-squared test revealed that the proportion of poor sleepers and individuals with EDS was greater in the ALS group compared to the control group (poor sleepers: *p* = 0.006; EDS: *p* = 0.009). The abovementioned results are shown in Table 1. In the binary logistic regression, ALS patients had higher total PSQI scores (*p* = 0.014) and ESS scores (*p* = 0.015) compared to the controls, after adjusting for sex, age, and BMI. Moreover, the percentage of individuals with poor sleep (*p* = 0.009) and EDS (*p* = 0.005) was also higher in the ALS patient group compared to the caregiver group. In terms of emotional problems, the HADS-anxiety/depression scores of the ALS patients were similar to those of their caregivers, and the prevalence of probable and definite anxiety and depression symptoms in patients with ALS did not differ from that observed in the caregivers (*p* > 0.05). The above results are shown in Table 2. The subgroup analyses revealed that female ALS patients demonstrated a significantly higher proportion of doubtful or definite anxiety compared to male patients (*p* = 0.036). Furthermore, advanced-stage patients (KCSS 3-4) exhibited significantly impaired sleep quality and severe daytime sleepiness, as indicated by higher PSQI and ESS scores, in comparison to early-stage patients (KCSS 1) (PSQI: *p* = 0.021; ESS: *p* = 0.031). Among the caregivers, female participants showed significantly poorer sleep than male participants (PSQI: *p* = 0.026; poor sleepers: *p* = 0.010). Detailed results are presented in Supplementary Tables 1–3.

**Table 2:** Differences in the non-motor symptoms between the patients with ALS and their caregivers.

**Non-motor symptoms**	**Univariate**		**Multivariate**	
	**OR (95% CI)**	***P*** **-value**	**OR (95% CI)**	***P*** **-value**
Pittsburgh Sleep Quality Index score	1.148 (1.030–1.278)	0.013	1.151 (1.029–1.289)	0.014
Poor sleeper (*n*/%)	2.619 (1.309–5.241)	0.007	2.664 (1.276–5.560)	0.009
Epworth Sleepiness Scale score	1.105 (1.006–1.213)	0.037	1.134 (1.025–1.254)	0.015
EDS	3.864 (1.328–11.244)	0.013	5.135 (1.640–16.072)	0.005
HADS-depression	1.053 (0.963–1.152)	0.254	1.061 (0.967–1.165)	0.207
Doubtful or definite depression	1.432 (0.679–3.018)	0.346	1.420 (0.657–3.066)	0.372
HADS-anxiety	1.083 (0.982–1.194)	0.110	1.095 (0.985–1.216)	0.092
Doubtful or definite anxiety	1.080 (0.501–2.325)	0.845	1.226 (0.543–2.767)	0.625

The analyses of the non-motor symptoms and ALS were performed using binary logistic regression. In the multivariate analysis, adjustments were made for age, sex, and body mass index. ALS, amyotrophic lateral sclerosis; EDS, excessive daytime sleepiness; HADS, Hospital Anxiety and Depression Scale. OR, odds ratio; 95% CI: 95% confidence interval.

### 3.3 Correlations between non-motor features and clinical information of patients with ALS

We performed an uncorrected multiple linear regression analysis of the data regarding non-motor symptoms and disease features of ALS. The results are shown in Supplementary Table 4. Based on the uncorrected results, the ALS features that were likely to be correlated with non-motor symptoms (*p* < 0.100 in univariate analysis) were further included in the regression analysis, along with sex, age, and BMI. We found that the PSQI score was negatively associated with the ALSFRS-R score (*p* = 0.032) and positively associated with the ΔFS score (*p* = 0.024). No associations between the ESS, HADS-depression score, or HADS-anxiety score and ALS features were found. The above results are shown in Table 3.

**Table 3:** Correlations between the non-motor symptoms and ALS features potentially associated with non-motor symptoms (p<0.100 in the univariate analysis).

**Non-motor symptoms**	**Disease duration (months)**		**Bulbar onset**		**ALSFRS-R score** ^**#**^		Δ**FS**^**#**^		**KCSS**		**FVC% pred** ^*^	
	β **(95% CI)**	***P*** **-value**	β **(95% CI)**	***P*** **-value**	β **(95% CI)**	***P*** **-value**	β **(95% CI)**	***P*** **-value**	β **(95% CI)**	***P*** **-value**	β **(95% CI)**	***P*** **-value**
PSQI score	/	/	/	/	−0.238 (−0.454, −0.022)	0.032	2.867 (0.397, 5.336)	0.024	0.294 (−1.015, 1.604)	0.654	−0.040 (−0.088, 0.009)	0.107
ESS score	/	/	−2.349 (−5.158, 0.461)	0.100	/	/	/	/	/	/	/	/
HADS-anxiety score	0.234 (−0.001, 0.470)	0.051	/	/	/	/	/	/	1.014 (−0.047, 2.074)	0.061	/	/
HADS-depression score	/	/	/	/	/	/	/	/	1.133 (−0.093, 2.358)	0.069	/	/

The analyses were performed using multiple linear regression, and the outcome was adjusted for age, sex, body mass index, and ALS features potentially associated with non-motor symptoms. ALS: amyotrophic lateral sclerosis; ALSFRS-R: ALS Functional Rating-Revised; ΔFS = (48-ALSFRS-R score)/disease duration; KCSS, King's College ALS staging system; FVC% pred, forced vital capacity as a percentage of the estimated value; PSQI, Pittsburgh Sleep Quality Index; ESS, Epworth Sleepiness Scale; HADS, Hospital Anxiety and Depression Scale. ^#^Due to the potential collinearity of the ALSFRS-R score and ΔFS, we analyzed them separately. ^*^Information on FVC% pred was available for sixty patients with ALS.

### 3.4 Correlation between sleep quality and plasma NfL

**Figure 1:**
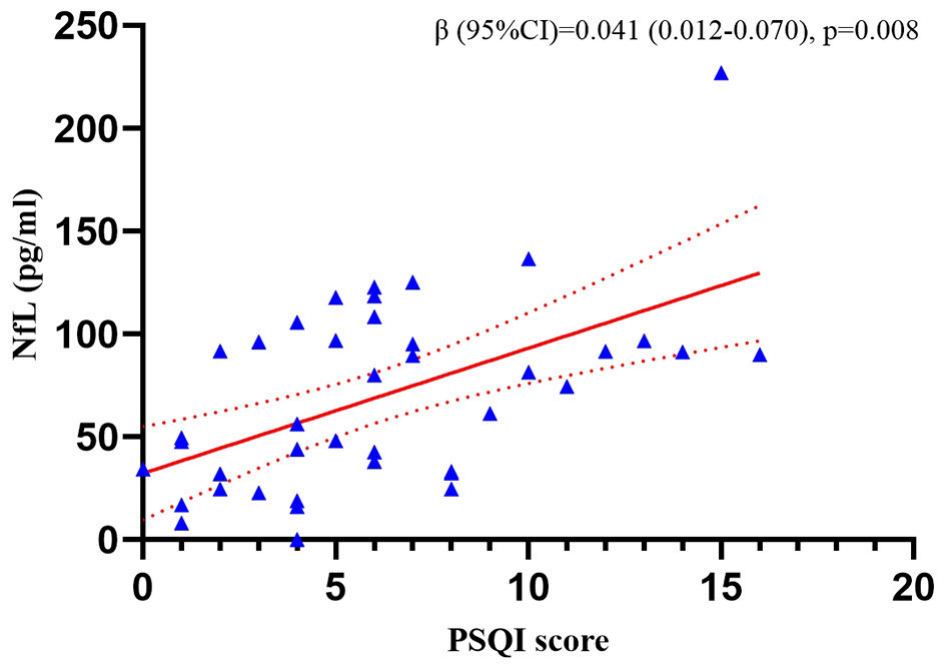
Correlation between the PSQI score and plasma NfL concentration in the patients with ALS. The analyses were performed using multiple linear regression. NfL, neurofilament light chain; PSQI, Pittsburgh Sleep Quality Index.

We tested NfL in 40 of the 69 patients from whom the plasma samples were collected. The multiple linear regression revealed that the PSQI score was positively correlated with the plasma NfL concentration (*p* = 0.008) after adjusting for age, sex, BMI, ALSFRS-R score, KCSS, and FVC% pred. The results are shown in Figure 1. Clinical information of the 40 patients is presented in Supplementary Table 5.

## 4 Discussion

In this clinical cross-sectional study, sleep quality, daytime sleepiness, anxiety, and depression in patients with early-stage ALS were evaluated using various scales and indices, and it was found that patients with early-stage ALS had poor sleep quality and daytime sleepiness. Moreover, we analyzed the correlation between these non-motor symptoms and the clinical features of ALS and found that low sleep quality was associated with more severe loss of motor function and more rapid disease progression.

Previous studies have reported significant sleep-related and emotional symptoms in ALS patients, but the patients included in these studies had a longer course of disease or more severe motor function impairment compared to those in our study (4, 5, 16). It remains unclear whether these non-motor symptoms are present during the early phase of the disease. By comparing the PSQI scores, we found that ALS patients in whom disease onset occurred within the previous 18 months had poorer sleep quality than their caregivers, consistent with the results obtained in the majority of previous studies (5). Moreover, we found that the ALSFRS-R score was positively associated with the PSQI score, suggesting that sleep quality is affected by the main symptoms of ALS even during the early stage of the disease when the condition is not yet severe. Furthermore, our analysis revealed that the patients in advanced disease stages (KCSS 3-4) had significantly higher PSQI scores compared to those in the initial stage (KCSS 1), providing additional evidence to support this potential association.

In the PSQI subproject, we found that sleep problems in ALS patients were mainly related to lower sleep efficiency, a finding similar to those reported in previous studies (5). However, at the same time, some of our findings conflict with those reported in previous studies. First, we did not find that ALS patients experience a delay in falling asleep. It has been reported that sleep delay in ALS patients is affected by restless legs syndrome (16). There is also evidence that RLS is closely associated with the preservation of lower limb function (16, 17). Thus, the relatively better motor function preservation in the patients included in our study may have contributed to the differences between our findings and those reported in earlier studies. Moreover, it has recently been reported that a high proportion of ALS patients experience limb paresthesia, a condition that may be related to autonomic nerve disorders, small-fiber neuropathy, and venous congestion of the limbs caused by the loss of motor function (18, 19). Paresthesia caused by these factors may also cause patients to have difficulty falling asleep. Our results suggest that autonomic dyskinesia and small-fiber neuropathy may not be severe in the early stages of the disease. Similarly, we did not observe sleep disturbances in the ALS patients from our study, and we think this may also be related to the reasons mentioned above.

We also explored the correlation between the non-motor symptoms and the clinical features of ALS patients. In the majority of previous studies, patients were grouped based on scale scores. However, considering that the PSQI and ESS have no recognized classification standards and that the grouping boundaries used in different studies are not consistent, we opted for a more secure approach, that is, the total scores on the two scales were treated as continuous variables. Surprisingly, we found that the PSQI score correlated positively with the ΔFS score, suggesting that patients with poor sleep quality might experience more rapid disease progression. To explore this possibility, we tested the plasma levels of NfL, an indicator that has been shown to correlate with the rate of ALS progression (20, 21), in some of the included patients. Further analysis confirmed the reliability of our findings.

There are several possible reasons that may explain our findings. First, poor sleep quality reflects pathological changes. In recent years, an increasing number of imaging studies have shown that changes in the brains of ALS patients are not limited to the motor system but also affect many structures outside the motor system, including the hypothalamus, thalamus, brainstem, and hippocampus (22–25); several of these structures are associated with sleep and wakefulness (26). Dedeene et al. performed pathological studies in which they showed that sleep-related neurons in the hypothalamus were damaged in ALS patients (27). In addition, Sun et al. reported that the sleep quality of ALS patients carrying disease-causing genes was lower than that of patients without these genes, a finding that also suggested that insomnia in ALS patients is related to pathological changes (28). Our previous animal experiments revealed that sleep disorders in SOD1G93A mice are related to the secretion of orexin by the hypothalamus (29). These findings strongly suggest that the sleep disturbances that occur in individuals with ALS may be related to pathological changes in areas of the central nervous system beyond the motor region. Moreover, a recent study revealed that ALS patients had higher endocannabinoidome levels compared to healthy controls and patients with other neurological diseases and that the endocannabinoidome is correlated with the rate of ALS progression (30). The endocannabinoidome also plays an important role in regulating sleep and wakefulness (31). The potential correlation between poor sleep quality and faster ALS progression suggested by our study may also depend on this pathway.

In addition, poor sleep quality and more rapid progression of ALS may be correlated because worsened sleep can accelerate the progression of ALS. In recent years, the glymphatic system has been found to be closely related to abnormal products in the central nervous system, and it has also been shown to be related to the progression of several neurodegenerative diseases (32, 33). The system operates more efficiently during sleep than wakefulness, a fact that mechanistically explains the relationship between neurodegenerative diseases and sleep (34). ALS has pathological similarities to common neurodegenerative diseases; therefore, it is possible that sleep disturbances promote ALS progression by inhibiting the function of the glymphatic system. In addition, poor sleep may accelerate the progression of ALS by promoting inflammatory responses (35).

We found that ALS patients had higher ESS scores and higher rates of EDS compared to their caregivers. This is consistent with the findings reported in previous studies and may also reflect pathological changes that occur in ALS patients (6). Whether patients with ALS experience emotional problems is a subject of controversy (36). The anxiety and depression levels of the ALS patients included in our study did not differ significantly from those of the caregivers. However, we cannot conclude that patients with early-stage ALS do not have emotional problems. Previous studies have reported that people who act as caregivers of ALS patients also have severe anxiety and depression (37). Furthermore, our findings revealed that female ALS patients exhibited significantly higher levels of anxiety compared to male patients, while the female caregivers demonstrated more pronounced sleep-related disturbances than the male caregivers. These results underscore the critical need for increased clinical attention to non-motor symptoms in both female patients and their female caregivers.

Our study has several limitations that have not been properly addressed. First, because cross-sectional studies only explore the correlations between various indicators, they provide evidence that may suggest conjectures and hypotheses rather than exact evidence. Further analysis of patient follow-up data is needed to verify the reliability of our findings. Second, during the long course of ALS, patients may take medications that affect their sleep. As discussed above, the paresthesia experienced by ALS patients may also affect their sleep. However, we were unable to comprehensively collect information on this issue and use it to explore these correlations. Our future studies will explore additional factors that may affect the sleep of ALS patients. Third, the number of patients in our analysis was small, and this might have resulted in selection bias. In future studies, we will attempt to increase the sample size. Fourth, the control group consisted of caregivers of ALS patients, who were not necessarily healthy, and their mood and sleep might have been affected by their individual conditions. Therefore, our results may not reliably reflect the differences in non-motor symptoms between ALS patients and healthy people. Future studies need to include healthy controls as a means of exploring this issue further. Moreover, caregivers of ALS patients bear a huge psychological burden and may experience sleep and emotional problems. Further research in this area would not only provide valuable insights but also potentially lead to the development of targeted interventions to support caregiver health. Fifth, the information we collected on non-motor symptoms was subjective and obtained through questionnaires, and no reliable evaluation of cognitive function was performed to assess whether the accuracy of the information was affected by cognitive dysfunction. Therefore, the reliability of the results was greatly limited. In future studies, we aim to obtain more reliable evidence on the relationship between non-motor symptoms and ALS by using more objective methods, such as polysomnographic monitoring, actigraphy, and comprehensive cognitive assessments. Sixth, our study focused exclusively on sleep-related and emotional aspects of non-motor symptoms in ALS patients, without investigating cognitive and behavioral abnormalities. Future research should expand the scope to include a more comprehensive range of non-motor symptoms.

Overall, we found higher proportions of poor sleepers and a higher incidence of EDS among the individuals with early ALS and that poor sleep quality was associated with more rapid progression of the disease in these patients. Our findings suggest that pathological changes that occur in the central nervous system during the early stage of ALS may already involve the motor system. This study provides new insights into understanding the pathological characteristics of ALS.

## Data Availability

The raw data supporting the conclusions of this article will be made available by the authors, without undue reservation.
