# Supplementary material for: Impact of sleep quality on disease progression in early-stage amyotrophic lateral sclerosis
Source: Front Neurol. 2025 Apr 10;16:1545463. doi: 10.3389/fneur.2025.1545463 (PMC12018231; doi:10.3389/fneur.2025.1545463)
Supplement: Supplementary file 1 [file Table_1.docx]

Supplementary table 1. Differences in nonmotor symptoms between male and female ALS patients.

|  | univariate | | multivariate | |
| --- | --- | --- | --- | --- |
|  | OR (95% CI) | *p* | OR (95% CI) | *p* |
| Pittsburgh Sleep Quality Index score | 1.046 (0.923, 1.185) | 0.484 | 1.049 (0.924, 1.190) | 0.460 |
| Poor sleeper (n/%) | 1.061 (0.395, 2.848) | 0.907 | 1.092 (0.402, 2,964) | 0.863 |
| Epworth Sleepiness Scale score | 0.925 (0.814, 1.052) | 0.234 | 0.915 (0.800, 1.047) | 0.197 |
| EDS | 0.750 (0.227, 2.476) | 0.637 | 0.733 (0.215, 2.500) | 0.620 |
| HADS-Depressive score | 1.050 (0.931, 1.184) | 0.423 | 1.051 (0.931, 1.187) | 0.424 |
| Doubtful or definite depression | 1.008 (0.352, 2.890) | 0.988 | 1.024 (0.353, 2.975) | 0.965 |
| HADS-Anxiety score | 1.140 (0.992, 1.309) | 0.065 | 1.148 (0.994, 1.325) | 0.060 |
| Doubtful or definite anxiety | 3.000 (0.991, 9.083) | 0.052 | 3.612 (1.085, 12.017) | 0.036 |

The analyses ere performed via binary logistic regression. In the multivariate analysis, adjustments were made for age and body mass index. ALS: amyotrophic lateral sclerosis; EDS: excessive daytime sleepiness; HADS: Hospital Anxiety and Depression Scale. OR: odds ratio; 95% CI: 95% confidence interval.
