# Supplementary material for: Impact of sleep quality on disease progression in early-stage amyotrophic lateral sclerosis
Source: Front Neurol. 2025 Apr 10;16:1545463. doi: 10.3389/fneur.2025.1545463 (PMC12018231; doi:10.3389/fneur.2025.1545463)
Supplement: Supplementary file 2 [file Table_2.docx]

Supplementary table 2. Differences in nonmotor symptoms between male and female caregivers of ALS patients.

|  | univariate | | multivariate | |
| --- | --- | --- | --- | --- |
|  | OR (95% CI) | *p* | OR (95% CI) | *p* |
| Pittsburgh Sleep Quality Index score | 1.269 (1.020, 1.580) | 0.033 | 1.297 (1.032, 1.629) | 0.026 |
| Poor sleeper (n/%) | 4.296 (1.420, 13.002) | 0.010 | 4.548 (1.447, 14.294) | 0.010 |
| Epworth Sleepiness Scale score | 1.024 (0.890, 1.178) | 0.742 | 1.031 (0.893, 1.189) | 0.679 |
| EDS | 1.597 (0.250, 10.208) | 0.621 | 1.809 (0.264, 12.404) | 0.546 |
| HADS-Depressive | 1.052 (0.915, 1.209) | 0.476 | 1.056 (0.917, 1.216) | 0.446 |
| Doubtful or definite depression score | 1.215 (0.406, 3.638) | 0.728 | 1.268 (0.413, 3.899) | 0.678 |
| HADS-Anxiety | 1.124 (0.968, 1.307) | 0.126 | 1.129 (0.966, 1.319) | 0.127 |
| Doubtful or definite anxiety score | 1.667 (0.550, 5.053) | 0.367 | 1.724 (0.562, 5.293) | 0.341 |

The analyses were performed via binary logistic regression. In the multivariate analysis, adjustments were made for age and body mass index. ALS: amyotrophic lateral sclerosis; EDS: excessive daytime sleepiness; HADS: Hospital Anxiety and Depression Scale. OR: odds ratio; 95% CI: 95% confidence interval.
