# Supplementary material for: Impact of sleep quality on disease progression in early-stage amyotrophic lateral sclerosis
Source: Front Neurol. 2025 Apr 10;16:1545463. doi: 10.3389/fneur.2025.1545463 (PMC12018231; doi:10.3389/fneur.2025.1545463)
Supplement: Supplementary file 3 [file Table_3.docx]

Supplementary table 3. Differences in nonmotor symptoms between ALS patients in difference KCSS stages.

|  | univariate | | multivariate | |
| --- | --- | --- | --- | --- |
|  | OR (95% CI) | *p* | OR (95% CI) | *p* |
| Pittsburgh Sleep Quality Index score |  |  |  |  |
| KCSS 1 | Reference | / | Reference | / |
| KCSS 2 | 1.131 (0.972, 1.316) | 0.112 | 1.108 (0.946, 1.297) | 0.204 |
| KCSS 3-4 | 1.230 (1.027, 1.474) | 0.024 | 1.249 (1.033, 1.510) | 0.021 |
| Poor sleeper |  |  |  |  |
| KCSS 1 | Reference | / | Reference | / |
| KCSS 2 | 1.653 (0.568, 4.809) | 0.356 | 1.464 (0.480, 4.467) | 0.503 |
| KCSS 3-4 | 2.100 (0.551, 8.002) | 0.277 | 2.264 (0.577, 8.880) | 0.241 |
| Epworth Sleepiness Scale score |  |  |  |  |
| KCSS 1 | Reference | / | Reference | / |
| KCSS 2 | 1.069 (0.929, 1.231) | 0.351 | 1.103 (0.944, 1.290) | 0.217 |
| KCSS 3-4 | 1.201 (1.012, 1.425) | 0.036 | 1.223 (1.018, 1.470) | 0.031 |
| EDS |  |  |  |  |
| KCSS 1 | Reference | / | Reference | / |
| KCSS 2 | 2.095 (0.548, 8.009) | 0.280 | 2.314 (0.555, 9.649) | 0.250 |
| KCSS 3-4 | 2.200 (0.456, 10.624) | 0.326 | 2.551 (0.489, 13.317) | 0.267 |
| HADS-Depressive score |  |  |  |  |
| KCSS 1 | Reference | / | Reference | / |
| KCSS 2 | 1.065 (0.927, 1.223) | 0.373 | 1.050 (0.904, 1.220) | 0.522 |
| KCSS 3-4 | 1.171 (0.992, 1.382) | 0.062 | 1.177 (0.995, 1.392) | 0.058 |
| Doubtful or definite depression |  |  |  |  |
| KCSS 1 | Reference | / | Reference | / |
| KCSS 2 | 2.965 (0.872, 10.079) | 0.082 | 2.991 (0.833, 10.744) | 0.093 |
| KCSS 3-4 | 2.333 (0.539, 10.098) | 0.257 | 2.481 (0.551, 11.161) | 0.236 |
| HADS-Anxiety score |  |  |  |  |
| KCSS 1 | Reference | / | Reference | / |
| KCSS 2 | 1.163 (0.987, 1.370) | 0.070 | 1.140 (0.957, 1.358) | 0.141 |
| KCSS 3-4 | 1.178 (0.974, 1.425) | 0.090 | 1.235 (0.996, 1.530) | 0.054 |
| Doubtful or definite anxiety |  |  |  |  |
| KCSS 1 | Reference | / | Reference | / |
| KCSS 2 | 2.211 (0.640, 7.639) | 0.210 | 1.675 (0.431, 6.513) | 0.457 |
| KCSS 3-4 | 1.145 (0.230, 5.711) | 0.868 | 1.706 (0.298, 9.756) | 0.548 |

The analyses were performed via multiple logistic regression. In the multivariate analysis, adjustments were made for age, sex and body mass index. ALS: amyotrophic lateral sclerosis; EDS: excessive daytime sleepiness; HADS: Hospital Anxiety and Depression Scale. OR: odds ratio; 95% CI: 95% confidence interval; KCSS: King's College staging system.
