# Supplementary material for: Impact of sleep quality on disease progression in early-stage amyotrophic lateral sclerosis
Source: Front Neurol. 2025 Apr 10;16:1545463. doi: 10.3389/fneur.2025.1545463 (PMC12018231; doi:10.3389/fneur.2025.1545463)
Supplement: Supplementary file 4 [file Table_4.docx]

Supplementary Table 4. Correlations between nonmotor symptoms and the clinical features of patients with ALS (univariate).

|  | Disease duration (months) | | Bulbar onset | | ALSFRS-R score | | ΔFS | | KCSS | | FVC% pred* | |
| --- | --- | --- | --- | --- | --- | --- | --- | --- | --- | --- | --- | --- |
|  | β (95% CI) | p | β (95% CI) | p | β (95% CI) | p | β (95% CI) | p | β (95% CI) | p | β (95%CI) | p |
| PSQI score | 0.149  (-0.098, 0.395) | 0.233 | 0.676  (-2.025, 3.378) | 0.619 | -0.360  (-0.530, -0.191) | <0.001 | 3.670  (1.860, 5.479) | <0.001 | 1.419  (0.285, 2.553) | 0.015 | -0.055  (-0.101, -0.009) | 0.021 |
| ESS score | -0.029  (-0.285, 0.227) | 0.824 | -2.817  (-5.510, -0.124) | 0.041 | -0.006  (-0.202, 0.190) | 0.949 | -0.377  (-2.447, 1.694) | 0.718 | 0.990  (-0.204, 2.183) | 0.103 | -0.002  (-0.052, 0.047) | 0.925 |
| HADS-Anxiety score | 0.270  (0.042, 0.498) | 0.021 | -1.920  (-4.456, 0.615) | 0.135 | -0.128  (-0.307, 0.052) | 0.160 | 0.760  (-1.154, 2.674) | 0.431 | 0.975  (-0.130, 2.079) | 0.083 | -0.020  (-0.065, 0.025) | 0.387 |
| HADS-Depressive score | 0.212  (-0.044, 0.467) | 0.103 | -0.603  (-3.426, 2.220) | 0.671 | -0.075  (-0.274, 0.124) | 0.455 | 0.194  (-1.914, 2.302) | 0.855 | 1.059  (-0.153, 2.270) | 0.086 | 0.010  (-0.039, 0.058) | 0.694 |

The analyses of the correlations between nonmotor symptoms and ALS features were performed via multiple linear regression, and the outcome was univariate. ALS: amyotrophic lateral sclerosis; ALSFRS-R: ALS Functional Rating-Revised; ΔFS=(48-ALSFRS-R score)/disease duration; KCSS: King's College ALS Staging System; FVC% pred: forced vital capacity as a percentage of the estimated value; PSQI: Pittsburgh Sleep Quality Index; ESS: Epworth Sleepiness Scale; HADS: Hospital Anxiety and Depression Scale; *Sixty patients with ALS had FVC% pred information.
