# Supplementary material for: Impact of sleep quality on disease progression in early-stage amyotrophic lateral sclerosis
Source: Front Neurol. 2025 Apr 10;16:1545463. doi: 10.3389/fneur.2025.1545463 (PMC12018231; doi:10.3389/fneur.2025.1545463)
Supplement: Supplementary file 5 [file Table_5.docx]

Supplementary Table 5. Characteristics of 40 patients with ALS who had NfL results.

|  | ALS patients (n=40) |
| --- | --- |
| Age (years) | 55.10±10.11 |
| Gender (n of male/%) | 22/55.00 |
| BMI | 24.02±3.14 |
| NfL (pg/ml) | 67.85 [32.59, 96.44] |
| Disease duration (months) | 9.62±3.17 |
| Bulbar onset (%) | 4/10.00 |
| FVC% pred | 84.10±19.17 |
| ALSFRS-R score | 43.00 [39.25 ,45.00] |
| ΔFS | 0.54 [0.35, 0.89] |
| KCSS (n/%) |  |
| 1 | 16/40.00 |
| 2 | 15/37.50 |
| 3 | 8/20.00 |
| 4 | 1/2.50 |
| HADS-anxiety | 6.00 [3.00,7.50] |
| HADS-depression | 6.00 [2.50, 8.00] |
| Pittsburgh Sleep Quality Index score | 6.00 [3.50, 8.00] |
| Subjective sleep quality | 1.00 [0.00, 1.75] |
| Sleep latency | 1.00 [0.00, 1.00] |
| Sleep duration | 1.00 [0.00, 2.00] |
| Habitual sleep efficiency | 1.00 [0.00, 1.00] |
| Sleep disturbances | 1.00 [1.00, 1.00] |
| Use of sleeping medication | 0.00 [0.00, 0.00] |
| Daytime dysfunction | 1.00 [0.00, 2.00] |
| Epworth Sleepiness Scale score | 5.00 [3.00, 9.00] |

ALS: amyotrophic lateral sclerosis; NfL: neurofilament light chain; BMI: body mass index; FVC: forced vital capacity as a percentage of the estimated value; ALSFRS-R: ALS Functional Rating–Revised; KCSS: King's College ALS staging system; HADS: Hospital Anxiety and Depression Scale; ΔFS=(48-ALSFRS-R score)/disease duration; *36 patients with ALS had accurate FVC% pred information.
